# Temperature-Dependent Food Consumption Rates of the Sea Urchin *Mesocentrotus nudus* and Top Shell *Turbo sazae*: Potential Impacts on Seaweed Beds

**DOI:** 10.3390/ani13223436

**Published:** 2023-11-07

**Authors:** Jaehwan Seo, Bon Joo Koo

**Affiliations:** 1 East Sea Environment Research Center, Korea Institute of Ocean Science & Technology (KIOST), Uljin 36315, Republic of Korea; playersjh@kiost.ac.kr; 2 School of Ocean Science, University of Science and Technology, Daejeon 34113, Republic of Korea

**Keywords:** *Mesocentrotus nudus*, *Turbo sazae*, food consumption rate, temperature, barren ground

## Abstract

**Simple Summary:**

Despite the expansion of barren grounds and a change in the habitats of macrograzers brought about by rising water temperatures linked to climate change, there is a lack of information regarding how this habitat alteration and grazing by macrograzers affect seaweed beds. This study assessed which species, sea urchin and top shell, would have a more significant influence on seaweed beds if the current trend of increasing water temperature continues. Our results suggest that top shell has a greater potential to cause barren ground and thus poses a greater threat than sea urchin.

**Abstract:**

In Korea, the expansion of barren ground and a shift in macrograzer habitats due to increasing water temperatures associated with climate change are becoming increasingly problematic. This study assessed the potential effects of the sea urchin *Mesocentrotus nudus* and top shell *Turbo sazae* on seaweed beds by examining changes in their food consumption rates in response to changes in temperature. The food consumption rates of kelp (*Saccharina japonica*) for both species were estimated at 5 °C, 10 °C, 15 °C, 20 °C, and 25 °C in laboratory experiments. The rate for *M. nudus* increased with increasing water temperature, with the highest rate of 0.001 g g^−1^ d^−1^ at 15 °C and 20 °C, and the lowest at 25 °C, which killed some individual sea urchins. The rate for *T. sazae* also increased with increasing water temperature, with the highest being 0.087 g g^−1^ d^−1^ at 25 °C and the lowest being at 5 °C. *T. sazae* had a higher food consumption rate than *M. nudus* at all temperatures; as water temperature increased, the difference between species increased, with the largest difference occurring at 25 °C. These findings indicate that as water temperature increases, *T. sazae* places greater feeding pressure on macroalgae than *M. nudus*.

## 1. Introduction

Urchin barren grounds are benthic communities on rocky reefs that are predominantly inhabited by herbivorous sea urchins and coralline red algae and that lack seaweed [1]. These barrens typically occur in areas capable of supporting kelp beds, which are highly productive, providing habitat and food sources for numerous ecologically and commercially significant fish and invertebrate species [2,3,4]. Over the past four decades, a shift from kelp beds to sea urchin barrens has been extensively documented along temperate coastlines worldwide [5,6,7,8]. In Korea, barrens initially emerged domestically in localized areas during the 1960s and 1970s [9,10], and their area of occurrence has been continuously expanding since then. Since the first reported occurrence of extensive barrens covering 370 ha in 1997, such areas have expanded significantly, reaching 2413 ha in 2003–2004 and a staggering 10,518 ha in 2014 along the eastern coast of Korea. Consequently, barrens are becoming an increasingly serious problem that affects 62% of the entire rock mass area of 170,054 ha [11].

High densities of herbivores, particularly sea urchins in the family Strongylocentrotidae, play a major role in the development of barren states in subtidal ecosystems [12,13,14,15]. In particular, the sea urchin *Mesocentrotus nudus* (formerly known as *Strongylocentrotus nudus*) is ecologically significant in coastal areas worldwide. This species is found in subtidal rocky regions across East Asia, including locations such as Dalian in China, the Maritime Territory of Russia, and the East Sea around Korea and Japan. This species is dominant in subtidal rocky habitats in Korea and typically favors brown algae such as *Saccharina longissima*, *S. japonica*, and *Undaria pinnatifida* as food resources [16,17,18]. Considering its high food consumption rate and population density, *M. nudus* has a substantial grazing impact on seaweed beds. This species is thought to be a significant contributor to the formation of barrens due to the overlap between its distribution and areas where seaweed has been depleted [19,20].

Since the mid-1970s, awareness of global climate change has increased; in particular, the impacts of increasing global seawater temperatures on ecological functions of marine species, such as range expansion or contraction and local extinction, have become clear [21,22,23]. The seas around the Korean Peninsula are some of the most vulnerable to climate change, as they are currently undergoing a rapid increase in seawater temperature [24]. Because Korean marine ecosystems extend from the sub-tropical to sub-arctic zones, a variety of marine organisms have likely shifted their geographical distributions poleward with recent global climate change [25]. In particular, during the past half century, increases in mean water temperature of nearly 1.7 °C have been reported in the East/Japan Sea, 1.4 °C in the Korea Strait, and 0.3 °C in the Yellow Sea [26].

Temperature influences the metabolism of marine ectotherm species and can thereby impact their behavior and reproductive success [27,28,29]. Many biological processes directly related to metabolic rates exhibit a unimodal performance curve when assessed across a suitable temperature range [30,31,32]. Within the temperature range of 0 °C to 40 °C, metabolic rates of ectotherms first increase almost proportionally with temperature, and then decrease rapidly above a critical temperature [27,31,32]. Furthermore, at higher water temperatures, marine ectotherms face reduced oxygen concentrations, limiting their capacity to feed or reproduce [32,33].

The Korean top shell, *Turbo sazae*, is a marine gastropod that inhabits sub-tropical rocky subtidal environments with water temperatures of 20 °C to 25 °C. This species is predominantly found in regions influenced by the Kuroshio–Tsushima current in the northwestern Pacific [34,35,36,37]. *T. sazae* has long been included within *Turbo cornutus* [38]. However, Fukuda [39] proposed that *T. cornutus* should refer only to populations that are native to southern China and Taiwan, designating the Japanese/Korean populations as a distinct species, *T. sazae*. Historically, *T. sazae* was primarily found along the coast of Jeju in Korean waters, with secondary populations along the coast of Busan [40]. Recent shifts in oceanographic conditions driven by climate change have led to northward expansion of the habitat range of *T. sazae*, from 35° N in 2010 to beyond the 37° N line in 2016, covering a distance of 124 km in just 6 years [41]. However, information about the effects of this habitat expansion and feeding by *T. sazae* on seaweed beds remains limited.

This study evaluated the potential impacts of the sea urchin *M. nudus* and top shell *T. sazae* on seaweed beds based on changes in their food consumption rates in response to changing temperature.

## 2. Materials and Methods

### 2.1. Animal Preparation

We obtained 50 sea urchins from the Imwon harbor fishery market in Samcheok, Korea, in September 2022. All individuals were immediately placed into containers filled with seawater and transported to the laboratory. Upon arrival, they were moved to aerated 60 L aquaria maintained at 15 °C. After 1 day, we selected 15 individuals exhibiting high mobility, rigid and upward-facing spines, and ambulacral feet that could adhere strongly to surfaces, thereby avoiding common signs of stress or disease. Each individual was transferred to an independent experimental unit and acclimated to the experimental temperature (5 °C, 10 °C, 15 °C, 20 °C, and 25 °C) without feeding for 5 days. The diameter and wet weight of each individual were measured immediately before and after the feeding assay.

In October 2022, 50 top shells were acquired from the fishery market at the Jukbyeon harbor in Uljin, Korea. The top shells were acclimated using the same method as sea urchins. The shell height and wet weight of each individual were measured immediately before and after the feeding assay. We selected a certain size of both species for the tests.

### 2.2. Experimental System

**Figure 1:**
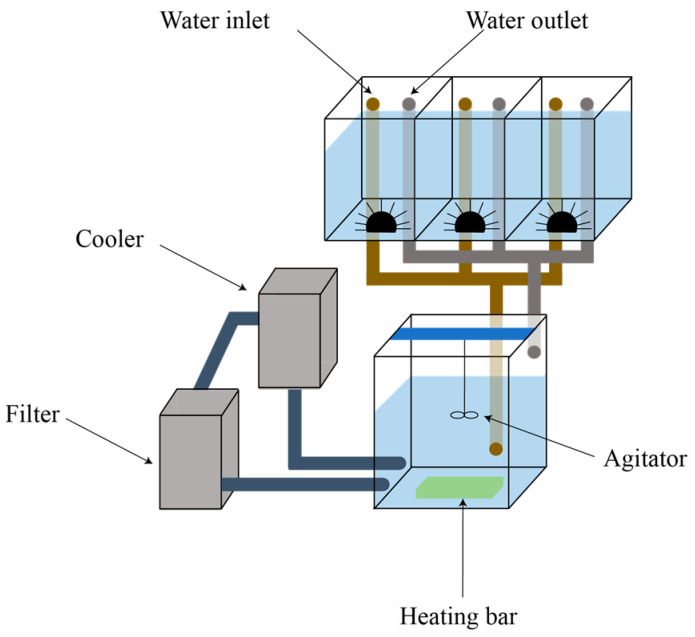
Schematic diagram of the experimental system used for the feeding assay with one main tank and three experimental units.

We manipulated seawater temperature using five main tanks from 5 °C to 25 °C at a 5 °C interval, and seawater was circulated from each main tank into three independent experimental units. A water pump and agitator were installed within each main tank to ensure homogenous mixing of the water in the main tanks and thus avoid the formation of temperature gradients. To adjust the water temperature, each main tank was equipped with electric heating and cooling units, which were controlled with electric temperature regulators. The regulators allowed the water temperature to be adjusted by a minimum interval of 0.2 °C. A temperature logger was placed within each main tank for continuous monitoring. A schematic diagram of the experimental system used for the feeding assay is presented in Figure 1.

The sea urchins and top shells were assigned to experimental units, each holding one individual of each species. These units were constructed of transparent polycarbonate (20 cm width × 30 cm height × 20 cm depth) with inflow and outflow holes at the top to maintain a constant water volume of 20 L. The experimental system was set up within a constant temperature and humidity chamber maintained at a temperature of 15 °C with a photoperiod of 12 h of light and 12 h of darkness.

Prior to the feeding assay, the sea urchins and top shells were acclimated to laboratory conditions for 1 day. During this time, they were kept at the sea surface temperature recorded during collection. Then, the water temperature was gradually increased or decreased at a rate of 5 °C per day for 3 days until the target temperature was reached. All animals were kept at the target water temperatures for 2 days, and were starved during the acclimation period.

### 2.3. Nutrient Analysis

Seawater samples were collected every 24 h from the main tank, transferred to polypropylene tubes, and stored at −80 °C for preservation. The concentrations of phosphate, nitrate, and nitrite were analyzed using a continuous flow analyzer (QuAAtro, SEAL Analytical, Mequon, WI, USA).

### 2.4. Feeding Assay and Consumption Rates

During the feeding assay, which lasted for 5 days, the sea urchins and top shells were fed kelp (*S. japonica*) of a specific size and weight (10 cm × 10 cm; wet weight: approximately 10 g). The kelp provided as food was replaced with fresh kelp every 24 h and this process was repeated over the entire feeding assay period. Additionally, three pieces of kelp were maintained as controls in the absence of animals to assess possible autogenic changes in kelp weight due to degradation or water saturation. The amount of kelp consumed by the sea urchins and top shells was determined based on the difference in the wet weight of kelp before and after the feeding assay, which was adjusted according to the change in kelp wet weight of controls. Then, we standardized the wet weight of consumed kelp to the wet weight of the animal and the duration of the feeding assay using the following equation:

Food consumption rate = *wet weight*_*kelp consumed*_ [mg]/(*wet weight*_*animal*_ [g] × *time* [d]) (1)

### 2.5. Statistical Analysis

Differences in the diameter and wet weight of sea urchins, and in shell height and wet weight of top shells, before and after the feeding assay were determined using a two-sample *t*-test. The results were considered statistically significant at *p* < 0.05. A one-way analysis of variance (ANOVA) with Tukey’s post-hoc test was used to assess differences in these measurements as well as food consumption rates at each temperature. All statistical analyses were performed using SYSTAT software (Systat version 12.0, SPSS Inc., Chicago, IL, USA).

## 3. Results

### 3.1. Comparison of Morphometric Data

The mean diameter and mean wet weight of *M. nudus* and *T. sazae* prior to the feeding assay showed no significant differences among temperatures (Table 1 and Table 2). The values for *M. nudus* only differed at 25 °C (Table 3). On the third day of the feeding assay at 25 °C, two sea urchins died in two experimental units, whereas no mortality of *T. sazae* was observed at any temperature. The values for *T. sazae* did not significantly differ before/after feeding or among temperatures (Table 4).

### 3.2. Seawater Nutrients

**Figure 2:**
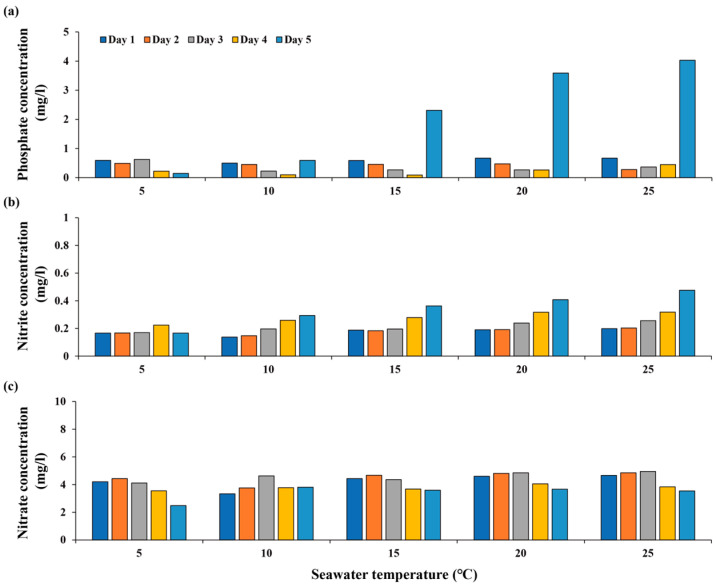
Comparison of nutrient concentrations in *M. nudus* treatments: (**a**) phosphate, (**b**) nitrite, and (**c**) nitrate.

The phosphate concentration in *M. nudus* treatments at 5 °C slightly decreased on the second day, followed by a slight increase on the fourth day and a sharp decrease on the fifth day, whereas it gradually decreased until the fourth day and then increased on the fifth day at 10 °C (Figure 2). It showed decreasing trends until the fourth day at 15 °C, 20 °C, and 25 °C, followed by rapid increases on the fifth day. The nitrite concentration gradually increased at all temperatures except 5 °C, while the nitrate concentration showed a consistent decreasing tendency over time.

**Figure 3:**
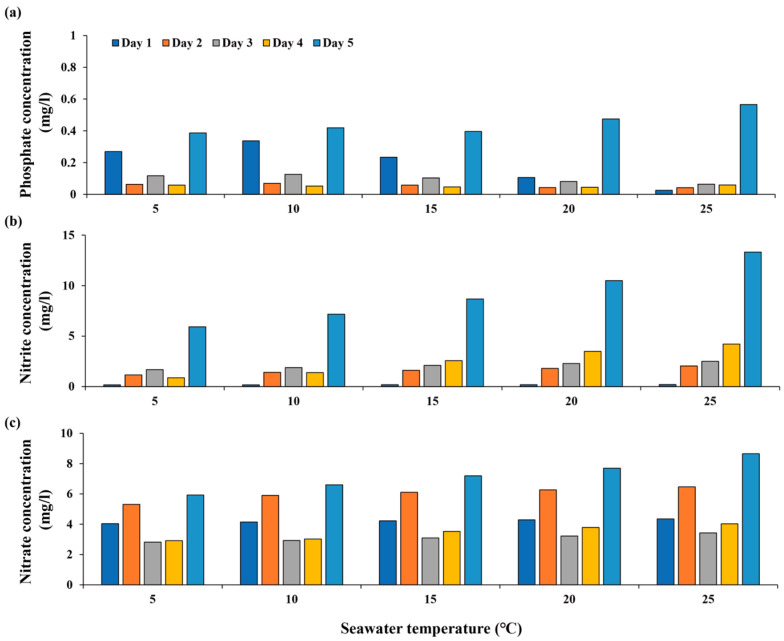
Comparison of nutrient concentrations in *T. sazae* treatments: (**a**) phosphate, (**b**) nitrite, and (**c**) nitrate.

In *T. sazae*, phosphate concentrations fluctuated until the fourth day at all temperatures except 25 °C, and rapidly increased on the fifth day at all temperatures (Figure 3). A rapid increase in nitrite was observed on the fifth day at all temperatures, while the nitrate concentration showed a fluctuating pattern with repeated decreases and increases.

### 3.3. Food Consumption Rates

**Figure 4:**
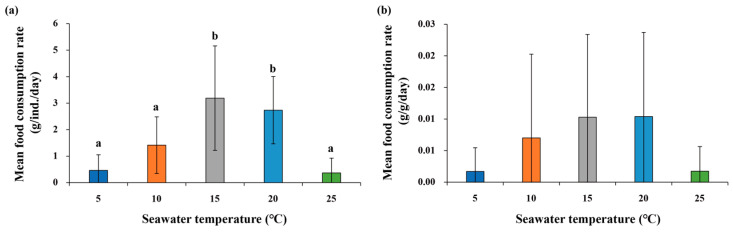
Comparison of food consumption rate of *M. nudus* by temperature: (**a**) food consumption rate per individual and (**b**) food consumption rate per unit biomass. Different letters indicate significant differences among temperatures at *p* < 0.05.

**Figure 5:**
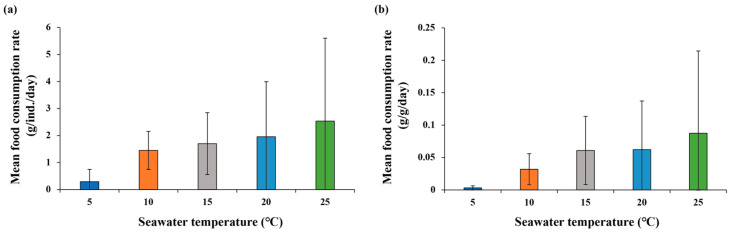
Comparison of food consumption rate of *T. sazae* by temperature: (**a**) food consumption rate per individual and (**b**) food consumption rate per unit biomass.

Variation in food consumption rates was observed in both *M. nudus* and *T. sazae* in response to changes in water temperature (Figure 4 and Figure 5). The mean rate per individual was highest at 15 °C, followed by 20 °C, 10 °C, 5 °C, and 25 °C. Meanwhile, the rate per unit biomass was highest at 15 °C and 20 °C, followed by 10 °C, 5 °C, and 25 °C.

For *T. sazae*, the rate increased with increasing water temperature. The rate per individual was highest at 25 °C, followed by 20 °C, 15 °C, 10 °C, and 5 °C; it showed a similar trend per unit biomass. These results indicate that *M. nudus* had a higher mean food consumption rate per individual at 15 °C and 20 °C than *T. sazae*, whereas the reverse pattern was observed at 25 °C; however, the rate per unit biomass was higher in *T. sazae* than in *M. nudus* at all temperatures, and the difference between the two species increased as temperature increased (Table 5).

## 4. Discussion

Differences in body size and body mass among macrograzers are important factors that influence their food consumption due to corresponding differences in energetic requirements and metabolic demands [42,43,44]. The mean diameter and wet weight of *M. nudus* and *T. sazae* did not significantly differ before and after the feeding assay or among temperatures before the assay. Thus, we assumed that factors such as body size and biomass did not influence the food consumption rate of either species.

Böttger et al. [45] reported that the food consumption rate and feces production of another sea urchin, *Lytechinus variegatus*, decreased with increasing phosphate concentration, particularly above 0.8 mg L^−1^. On the other hand, an increase in food consumption by the sea urchin *Loxechinus albus* was observed with increasing phosphate concentration, suggesting that the effect of phosphate concentration on food consumption is species-specific [46]. Nitrate and nitrite are toxic to invertebrates, as they hinder the capacity of pigments to carry oxygen [47,48]. In addition, increases in their concentrations have been shown to lower the amount of food eaten by other gastropods [49]. Basuyaux and Mathieu [50] reported that the food consumption rate of the sea urchin *Paracentrotus lividus* significantly decreased when nitrate and nitrite concentrations exceeded 100 mg L^−1^ and 2 mg L^−1^, respectively. Over the entire feeding assay period of our study, these concentrations did not exceed those levels, suggesting that the food consumption rate of *M. nudus* was not influenced by these substances.

In *T. sazae*, the nitrite concentrations rapidly increased on the fifth day at all temperatures, reaching 13 mg L^−1^ at 25 °C, higher than the levels observed in *M. nudus* treatments (Figure 3). The feces of gastropods may contain high nitrogen levels due to low assimilation efficiencies for certain food sources [51]. Thus, we ascribed this change in nitrite to dissolution from feces. Watson et al. [52] reported that nitrite tolerance is species-specific, with species of the genus *Turbo* having higher tolerance to nitrite than other genera. They reported that mortality of two turbinid gastropods, *Tectus fenestratus* and *Tegula eiseni*, was observed at low concentrations, whereas no adverse effects were observed in *Turbo bruneus* at concentrations up to 5 mg L^−1^. In our study, the nitrite levels exceeded 5 mg L^−1^ on the fifth day at all temperatures, suggesting that food consumption may have been affected by excess nitrite. However, because nitrite tolerance varies among species [47,49,52], the concentration that impacts the food consumption rate of this species remains unclear. Therefore, further research is needed to assess the effects of seawater nutrient concentrations on the food consumption rates of *T. sazae*.

Regarding mortality, in *M. nudus*, there were no deaths except at 25 °C, consistent with a previous study that reported no mortality at a temperature range of 5–24 °C [53]. The food consumption rate of this species gradually increased from 5 °C to 15 °C, remained relatively constant from 15 °C to 20 °C, and then sharply decreased at 25 °C, in line with a previous study [54]. Thus, the rates were lowest at 5 °C and 25 °C, which may be attributable to limitations of effective grazing behaviors (i.e., the use of tube feet and spines to capture food and the lantern to grasp and masticate), reduced activity of digestive enzymes, and decreased nutrient absorption under conditions of temperature-induced stress [55,56,57,58]. Our findings suggest that the optimal temperature range for food consumption by *M. nudus* is between 15 °C and 20 °C. In contrast to *M. nudus*, no mortality of *T. sazae* was observed during the experiment. *T. sazae* is a sub-tropical species found in subtidal habitats with water temperatures of 14 °C to 25 °C [35,37], which suggests that it can withstand higher temperatures than *M. nudus*. In our study, it showed a gradual increase in the food consumption rate with an increase in temperature, with a peak at 25 °C. In such gastropods, increases in temperature lead to higher metabolic demand, thus increasing food consumption [59,60,61,62]. For example, the rate of *Turbo samaticus* gradually increases with increasing temperature, peaking at 25 °C [63]; in *Haliotis midae*, the rate doubles at 15 °C compared to 22 °C [64]. Furthermore, the oxygen consumption of *T. sazae* (*T. cornutus*) showed a gradual increase with increasing temperature, peaking at 30 °C, after which it rapidly declines [65]. Therefore, we conclude that this is the case in *T. sazae*. Such temperature-related changes in food consumption differed between the two species we studied. *M. nudus* had its highest food consumption rates at 15 °C and 20 °C, whereas *T. sazae* peaked at 25 °C. In addition, the difference in rate per unit biomass widened with increasing temperature. This suggests that as water temperature increases, *T. sazae* exerts greater feeding pressure on macroalgae than *M. nudus*. However, the feeding rates of macrograzers are also influenced by various factors other than temperature, including size, biomass, density, and food preference.

Because metabolic energy demands increase more rapidly with increasing body size than the amount of energy that can be supplied via consumption, and this mismatch is enhanced at elevated temperatures, the food consumption rates of sea urchins decrease as their size increases [32,44]. In contrast, the food consumption rates of turbinid gastropods show positive correlations with size, biomass, and density [42,66]. Food preference is one of the most important factors determining the food consumption rates of macrograzers. Yang et al. [20] reported that *M. nudus* preferred the brown alga *U. pinnatifida*, followed by *Grateloupia elliptica* and *Sargassum confusum*, with the consumption rate of the former being approximately double those of the other two. The high absorption efficiency of brown kelp by sea urchins is due to a high level of digestive enzymes for depolymerization of alginic acid and mannitol [67,68]. In contrast to sea urchins, turbinid gastropods have a preference for blue-green algae. For example, *Turbo marmoratus* primarily eats *Lyngbya* and *Rivularia* species, followed by coralline algae such as Melobesioideae and *Jania* species [69]. Despite the high abundance of the brown alga *Dictyopteris undulata* in its habitat, it does not consume it due to defensive chemicals such as sulfur compounds [20,69]. Hence, *M. nudus* and *T. sazae* probably have different preferred food sources.

Previous feeding conditions and changes in temperature can alter the food preferences of macrograzers; they tend to prefer foods that are different from what they have already consumed. For example, in one study, when *M. nudus* was starved before feeding, they preferred *U. pinnatifida*; in contrast, when fed this species and then tested, they preferred *G. elliptica* [20]. In another study, the green sea urchin *Strongylocentrotus droebachiensis* preferred *S. latissima* over *Codium fragile* but this changed depending on past feeding conditions [70]. They attributed the shift in preference to the benefits of a mixed diet, a need for different nutrients, and an attempt to avoid the accumulation of defensive chemicals [71,72,73,74,75]. The food preference of the turbinid gastropod *T. sarmaticus* is influenced by changes in temperature [63]. In the cited study, among six algal diets tested, *T. sarmaticus* consumed more *Ulva rigida* at 15 °C but more *Corallina* at 20 °C and 25 °C. The higher specific consumption rates may well equally be a result of compensation by *T. sarmaticus* for the lower digestibility of these algae. With all of this in mind, our study may be limited in that we offered only one species of food and thus our results may be overestimates or underestimates. Further research is required to determine the precise food consumption rates of these two species while considering their different food preferences.

The sea urchin *M. nudus* is one of the most abundant macrograzer species along the eastern coast of Korea, with population densities of 1 to 12 ind^−1^ m^−2^ [76]. It is believed to significantly contribute to the formation of barren ground due to overlap between its distribution and areas where seaweed has been depleted [19,20]. Recently, the habitat of *T. sazae* has expanded northward as seawater temperature has increased due to climate change; its population density has also increased [41]. However, information about the impacts of feeding by this species on seaweed beds remains limited. Our findings suggest that if the current trend of increasing water temperature continues, the potential impact of *T. sazae* on seaweed beds may become greater than the threat from *M. nudus*.

## 5. Conclusions

We evaluated changes in the food consumption rates of the sea urchin *M. nudus* and top shell *T. sazae* with changes in temperature. *T. sazae* had a higher consumption rate than *M. nudus* at all temperatures and the difference between species became wider as temperature increased. Given the ongoing range expansion of *T. sazae* due to increasing water temperature, our findings indicate that this species has a greater potential to cause barren ground and thus poses a greater threat than *M. nudus*. However, our study had limitations, as we did not consider various factors that may influence the feeding rates of both species. Nonetheless, our findings could help inform management plans for seaweed beds and strategies for restoration of barren grounds.

## Figures and Tables

**Table 1:** Comparison of morphometric data for *M. nudus* among temperatures prior to the feeding assay (mean ± standard deviation).

	5 °C	10 °C	15 °C	20 °C	25 °C
Diameter (mm)	64.8 ± 2.7	65.6 ± 7.4	66.6 ± 6.6	62.4 ± 2.2	66.5 ± 6.1
Wet weight (g)	91.1 ± 8.1	88.8 ± 30.0	93.5 ± 27.5	78.9 ± 8.2	92.6 ± 24.4

**Table 2:** Comparison of morphometric data for *T. sazae* among temperatures prior to the feeding assay (mean ± standard deviation).

	5 °C	10 °C	15 °C	20 °C	25 °C
Shell height (mm)	75.0 ± 2.7	77.2 ± 1.1	75.5 ± 6.4	70.3 ± 0.3	73.7 ± 3.9
Wet weight (g)	91.8 ± 8.2	101.5 ± 2.5	91.6 ± 14.5	84.6 ± 1.3	89.8 ± 18.2

**Table 3:** Comparison of morphometric data for *M. nudus* among temperatures and between measurements before and after the feeding assay (mean ± standard deviation). Significant differences are based on *t*-test at *p* < 0.05.

	5 °C		10 °C		15 °C		20 °C		25 °C	
	Diameter (mm)	Wet Weight (g)	Diameter (mm)	Wet Weight (g)	Diameter (mm)	Wet Weight (g)	Diameter (mm)	Wet Weight (g)	Diameter (mm)	Wet Weight (g)
Before	64.8 ± 2.7	94.1 ± 8.1	65.6 ± 7.4	88.8 ± 30.0	66.6 ± 6.6	93.5 ± 27.5	62.4 ± 2.3	78.9 ± 8.3	67.7	102.4
After	66.0 ± 2.7	94.1 ± 8.7	67.8 ± 8.2	87.8 ± 37.0	67.7 ± 5.3	91.6 ± 22.8	64.1 ± 3.0	78.3 ± 3.9	70.3	102.3
*p* value	0.05<	0.05<	0.05<	0.05<	0.05<	0.05<	0.05<	0.05<	nd	nd

nd: not determined.

**Table 4:** Comparison of morphometric data for *T. sazae* among temperatures and between measurements before and after the feeding assay (mean ± standard deviation). Significant differences are based on *t*-test at *p* < 0.05.

	5 °C		10 °C		15 °C		20 °C		25 °C	
	Diameter (mm)	Wet Weight (g)	Diameter (mm)	Wet Weight (g)	Diameter (mm)	Wet Weight (g)	Diameter (mm)	Wet Weight (g)	Diameter (mm)	Wet Weight (g)
Before	64.9 ± 2.9	91.8 ± 8.2	67.8 ± 0.8	101.5 ± 2.5	65.8 ± 3.4	91.6 ± 14.5	70.3 ± 0.3	84.6 ± 1.3	73.7 ± 3.9	89.8 ± 18.2
After	65.5 ± 2.3	87.8 ± 6.4	67.6 ± 0.8	100.3 ± 2.9	66.0 ± 3.4	91.2 ± 14.1	70.5 ± 0.57	84.7 ± 1.6	7.3 ± 4.4	90.0 ± 18.5
*p* value	0.05<	0.05<	0.05<	0.05<	0.05<	0.05<	0.05<	0.05<	0.05<	0.05<

**Table 5:** Comparison of food consumption rate between *M. nudus* and *T. sazae* (mean ± standard deviation). Significant differences are based on *t*-test at *p* < 0.05.

	5 °C		10 °C		15 °C		20 °C		25 °C	
	Ind. (g ind^−1^ d^−1^)	Biomass (g g^−1^ d^−1^)	Ind. (g ind^−1^ d^−1^)	Biomass (g g^−1^ d^−1^)	Ind. (g ind^−1^ d^−1^)	Biomass (g g^−1^ d^−1^)	Ind. (g ind^−1^ d^−1^)	Biomass (g g^−1^ d^−1^)	Ind. (g ind^−1^ d^−1^)	Biomass (g g^−1^ d^−1^)
*M. nudus*	0.46 ± 0.59	0.002 ± 0.004	1.41 ± 1.07	0.007 ± 0.013	3.19 ± 1.97	0.010 ± 0.013	2.73 ± 1.27	0.010 ± 0.013	0.37 ± 0.56	0.002 ± 0.004
*T. sazae*	0.29 ± 0.46	0.003 ± 0.003	1.45 ± 1.70	0.032 ± 0.024	1.70 ± 1.14	0.061 ± 0.053	1.96 ± 2.04	0.062 ± 0.075	2.54 ± 3.07	0.087 ± 0.127
*p* value	0.05<	0.05<	0.05<	0.05<	0.05<	0.05<	0.05<	0.05<	0.05<	0.05<

## Data Availability

Data are contained within the article.
